# Predictors of Successful Flap Reconstruction for Pressure Ulcers: A Retrospective Analysis of Patient and Ulcer Characteristics

**DOI:** 10.7759/cureus.111562

**Published:** 2026-06-26

**Authors:** Aakash Pandey, Yash Shrivastava, Prashant, Amber Yadav

**Affiliations:** 1 General Surgery, LN Medical College and Research Center and JK Hospital, Bhopal, IND; 2 Surgical Gastroenterology, All India Institute of Medical Sciences, Bhopal, Bhopal, IND; 3 Plastic and Reconstructive Surgery, LN Medical College and Research Center and JK Hospital, Bhopal, IND

**Keywords:** fasciocutaneous flap, flap reconstruction, postoperative complications, pressure sore, pressure ulcer, wound healing

## Abstract

Background

Pressure ulcers are a major cause of morbidity in immobilized and debilitated patients. Advanced pressure ulcers often require flap reconstruction for durable wound coverage; however, postoperative complications and recurrence remain significant concerns. Identification of factors influencing flap survival and wound healing is important for improving surgical outcomes.

Aim

This study aimed to identify predictors of successful flap reconstruction in patients undergoing surgical management for pressure ulcers.

Materials and methods

A retrospective observational study was conducted in the Department of General Surgery, LN Medical College and JK Hospital, Bhopal, from November 2024 to November 2025. Twenty patients with Stage III and Stage IV pressure ulcers who underwent flap reconstruction were included. Patient-related factors, including age, sex, nutritional status, anemia, and comorbidities, were recorded. Ulcer-related variables, including site, stage, duration, infection, and osteomyelitis, were also analyzed. Different flap procedures, including the superior gluteal artery perforator (SGAP) flap, tensor fascia lata flap, bilateral V-Y advancement flap, and rotational flap, were performed according to ulcer characteristics. Postoperative outcomes, including flap survival, wound healing, complications, and recurrence, were assessed.

Results

The mean age of patients was 58.9 ± 10.6 years, with male predominance, accounting for 12 (60.0%) patients. Sacral ulcers were the most common ulcers observed, noted in 10 (50.0%) patients, followed by trochanteric ulcers in five (25.0%) patients. Most ulcers were Stage IV pressure ulcers, seen in 12 (60.0%) patients. Successful flap healing was achieved in all patients, with no recurrence noted during follow-up. Postoperative complications were observed in five (25.0%) patients and included seroma formation, wound dehiscence, partial flap necrosis, and abscess formation. Poor nutritional status (p = 0.01), anemia (p = 0.04), infection/osteomyelitis (p = 0.002), and prolonged ulcer duration (p = 0.03) were significantly associated with postoperative complications.

Conclusion

Flap reconstruction provides effective management for advanced pressure ulcers with favorable healing outcomes. Optimization of nutritional status, infection control, pressure off-loading, and management of comorbidities are essential factors that influence flap survival and reduce postoperative complications.

## Introduction

Pressure ulcers are localized injuries to the skin and underlying tissues that usually occur over bony prominences due to prolonged pressure, shear, and friction, and are often associated with impaired mobility. They are commonly seen in elderly, bedridden, neurologically impaired, and paraplegic patients and are associated with significant morbidity, prolonged hospitalization, and increased healthcare costs [1,2]. Advanced pressure ulcers, particularly Stage III and IV ulcers, often require surgical management because conservative treatment alone is insufficient for wound healing. Surgical flap reconstruction remains the preferred treatment modality as it provides durable, well-vascularized tissue coverage and reduces the risk of recurrence [3].

Various reconstructive options, including fasciocutaneous, musculocutaneous, rotational, V-Y advancement, tensor fascia lata (TFL), and perforator-based flaps, are used depending on the site and severity of the ulcer [4]. Although flap reconstruction has improved outcomes in pressure ulcer management, postoperative complications such as wound dehiscence, seroma, hematoma, flap necrosis, infection, and ulcer recurrence continue to remain major challenges [5]. Several studies have reported recurrence rates ranging from 3% to more than 50%, depending on patient characteristics, ulcer severity, comorbid conditions, and duration of follow-up [6].

Successful flap reconstruction depends not only on the surgical technique but also on multiple patient-related and ulcer-related factors. Poor nutritional status, anemia, uncontrolled diabetes mellitus, infection, osteomyelitis, prolonged immobilization, bladder and bowel incontinence, and large, long-standing ulcers have been identified as important predictors of poor healing and recurrence [7]. Similarly, inadequate pressure off-loading, poor postoperative care, and noncompliance with rehabilitation protocols may adversely affect flap survival and long-term outcomes [8].

Preoperative optimization of the patient, proper wound bed preparation, control of infection, and careful selection of flap type are essential for successful reconstruction. Identification of factors associated with successful flap healing can help surgeons improve patient selection, reduce complications, and optimize postoperative outcomes. However, there is limited data from Indian tertiary care centers regarding predictors influencing flap success in pressure ulcer reconstruction. Therefore, the present study was undertaken to retrospectively analyze patient and ulcer characteristics associated with successful flap reconstruction in pressure ulcers. The study also aims to evaluate postoperative outcomes and identify factors influencing flap survival, wound healing, complications, and recurrence.

## Materials and methods

Study design and setting

A retrospective observational study was conducted in the Department of General Surgery at LN Medical College and JK Hospital, Bhopal, over a period of one year, from November 2024 to November 2025. The study was undertaken to evaluate predictors of successful flap reconstruction in patients with pressure ulcers. Patients with Stage III and Stage IV pressure ulcers who underwent flap reconstruction during the study period were included. A total of 20 patients were analyzed.

Eligibility criteria

Patients with Stage III and Stage IV pressure ulcers who underwent flap reconstruction surgery were included in the study. Patients with cardiopulmonary disease, those receiving chemotherapy, and those with previously operated scars that interfere with flap planning or harvest were excluded from the study.

Data collection

Data were collected retrospectively from hospital records, operative notes, and follow-up records using a structured data collection proforma. Demographic and clinical variables, including age, sex, nutritional status, anemia, and associated comorbidities, were recorded. Ulcer-related characteristics, including site, size, stage, ulcer duration, and presence of infection or osteomyelitis, were also documented. Surgical details regarding the type of flap used for reconstruction were noted.

Surgical procedure

All surgical procedures were performed under general or regional anesthesia with the patient positioned prone for sacral and ischial defects or in a lateral decubitus position for trochanteric lesions. The operational workflow was uniformly divided into two meticulous phases consisting of radical wide debridement followed by tailored soft-tissue reconstruction. During the initial phase, the visible margins of the ulcer were outlined, and intravenous prophylactic antibiotics were administered thirty minutes prior to the skin incision. Meticulous surgical debridement of all necrotic tissues, high-burden bacterial bioburden, infected bursa, and unviable fascial layers was executed until healthy, actively bleeding margins were reached. In cases with clinical or radiological signs of osseous involvement, partial ostectomy or bone curettage was performed to obtain fragments for histopathological evaluation and microbiological culture. Following debridement, the open wound bed was copiously irrigated with normal saline and an aqueous povidone-iodine solution, after which surgical drapes and instruments were completely changed to prevent cross-contamination during the subsequent reconstructive phase.

**Figure 1:**
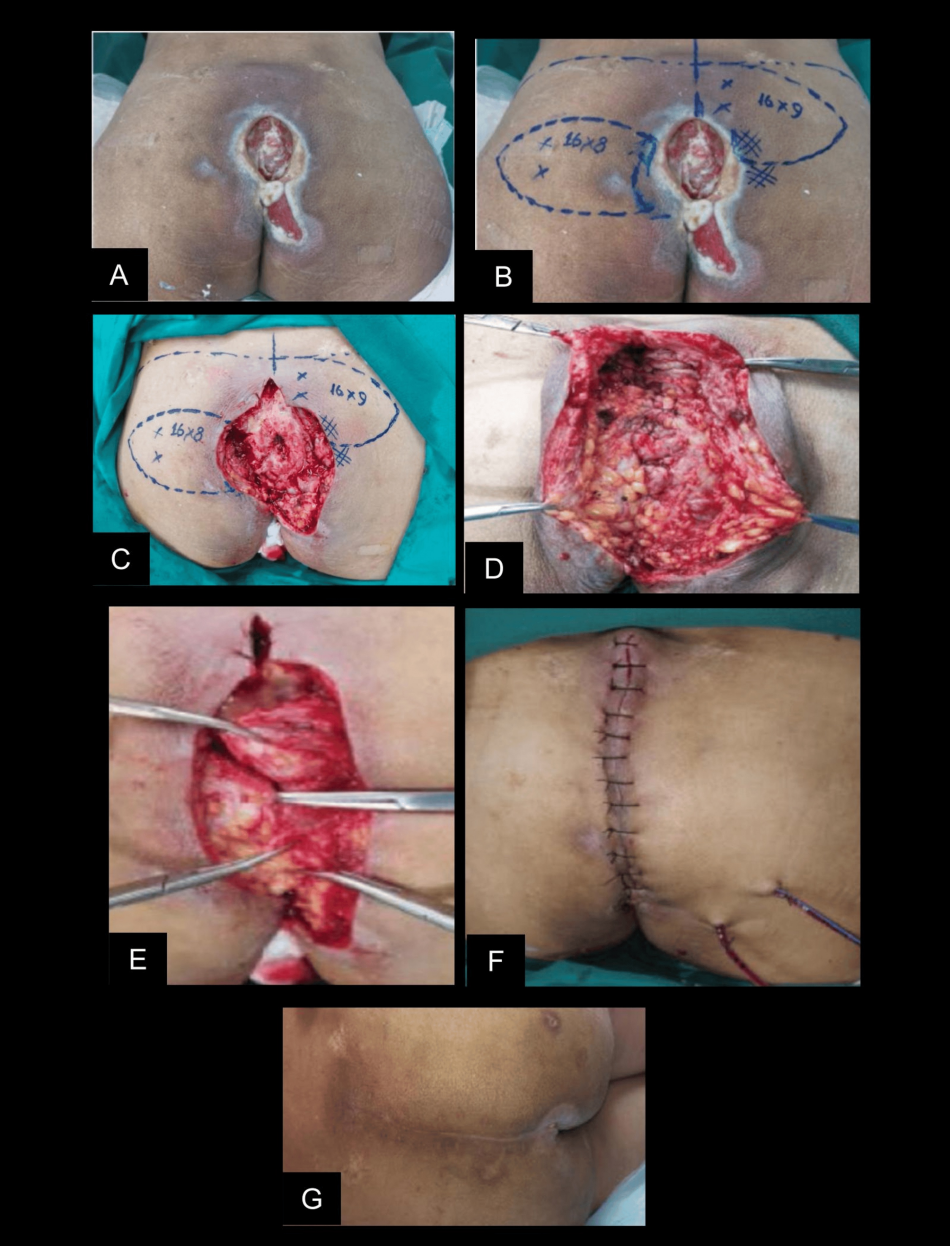
Comprehensive sequential operative stages of an advanced sacral pressure ulcer reconstruction using a bilateral V-Y advancement flap. (A) Preoperative presentation of a severe, full-thickness Stage IV sacral pressure ulcer with localized tissue breakdown extending into the natal cleft. (B) Intraoperative planning and marking of the bilateral V-Y advancement fasciocutaneous flaps, with precise dimensions (16 × 8 cm and 16 × 9 cm) outlined symmetrically on the gluteal skin surfaces adjacent to the lesion. (C) Intraoperative view displaying the initial surgical incision along the mapped margins following the completion of radical wide debridement of the ulcer bed. (D) Deep exploration and meticulous mobilization of the bilateral fasciocutaneous tissue islands, ensuring adequate perforator preservation for flap viability. (E) Mobilization and medial advancement of the flap edges toward the midline defect to achieve primary, tension-free closure. (F) Immediate postoperative outcome following definitive flap inset and wound closure along the midline in a classic V-Y configuration, supported by two closed-suction surgical drains placed in situ. (G) Long-term clinical follow-up, demonstrating complete, stable wound healing, robust soft-tissue coverage over the sacral prominence, and well-matured surgical scars with no signs of ulcer recurrence.

Flap architecture was individualized based on the specific anatomical location, defect dimensions, wound geometry, and the availability of surrounding donor tissue. For large, deep midline sacral defects, bilateral V-Y advancement flaps were frequently utilized. The clinical sequence began with the preoperative identification of the extensive sacral tissue breakdown (Figure 1A). Symmetrical, triangular fasciocutaneous margins were then mapped adjacent to the wound bed (Figure 1B) and incised down to the gluteal fascia (Figure 1C). These flaps were carefully mobilized while strictly preserving the underlying musculocutaneous perforators to maintain tissue viability (Figure 1D) before being advanced medially toward the midline (Figure 1E). Secure deep fascial anchoring was performed to eliminate dead space, and cutaneous edges were approximated in a classic configuration over two active closed-suction surgical drains placed in situ (Figure 1F), culminating in stable long-term wound healing with no signs of recurrence (Figure 1G).

**Figure 2:**
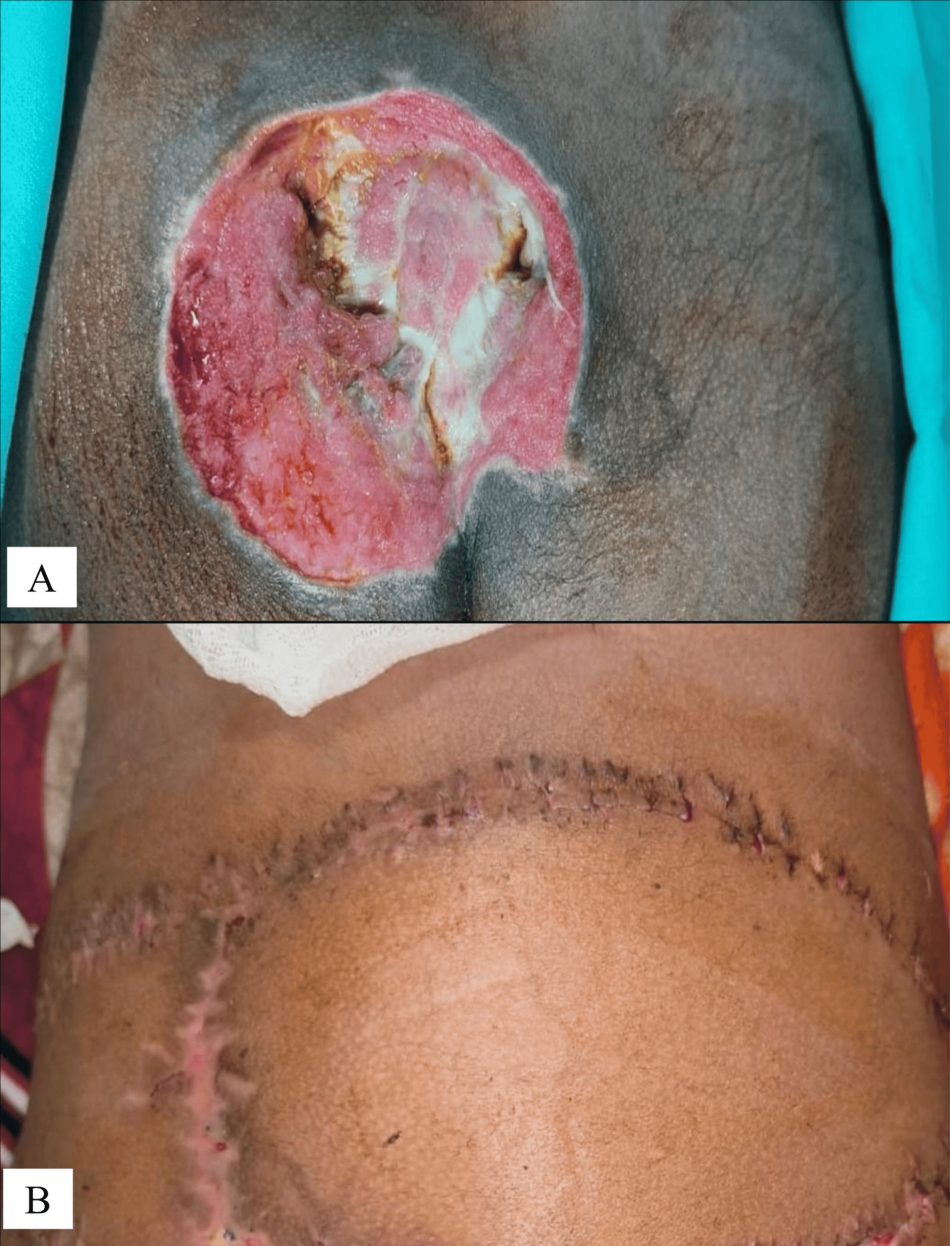
Reconstructive sequence of an advanced sacral pressure ulcer utilizing a superior gluteal artery perforator (SGAP) flap. (A) Preoperative presentation of a large, full-thickness Stage IV sacral pressure ulcer exhibiting a deep central crater, fibrotic margins, and prominent central slough. (B) Late postoperative clinical follow-up demonstrating complete, stable healing of the transposed perforator flap with well-matured surgical scars and robust soft tissue padding over the sacral prominence.

**Figure 3:**
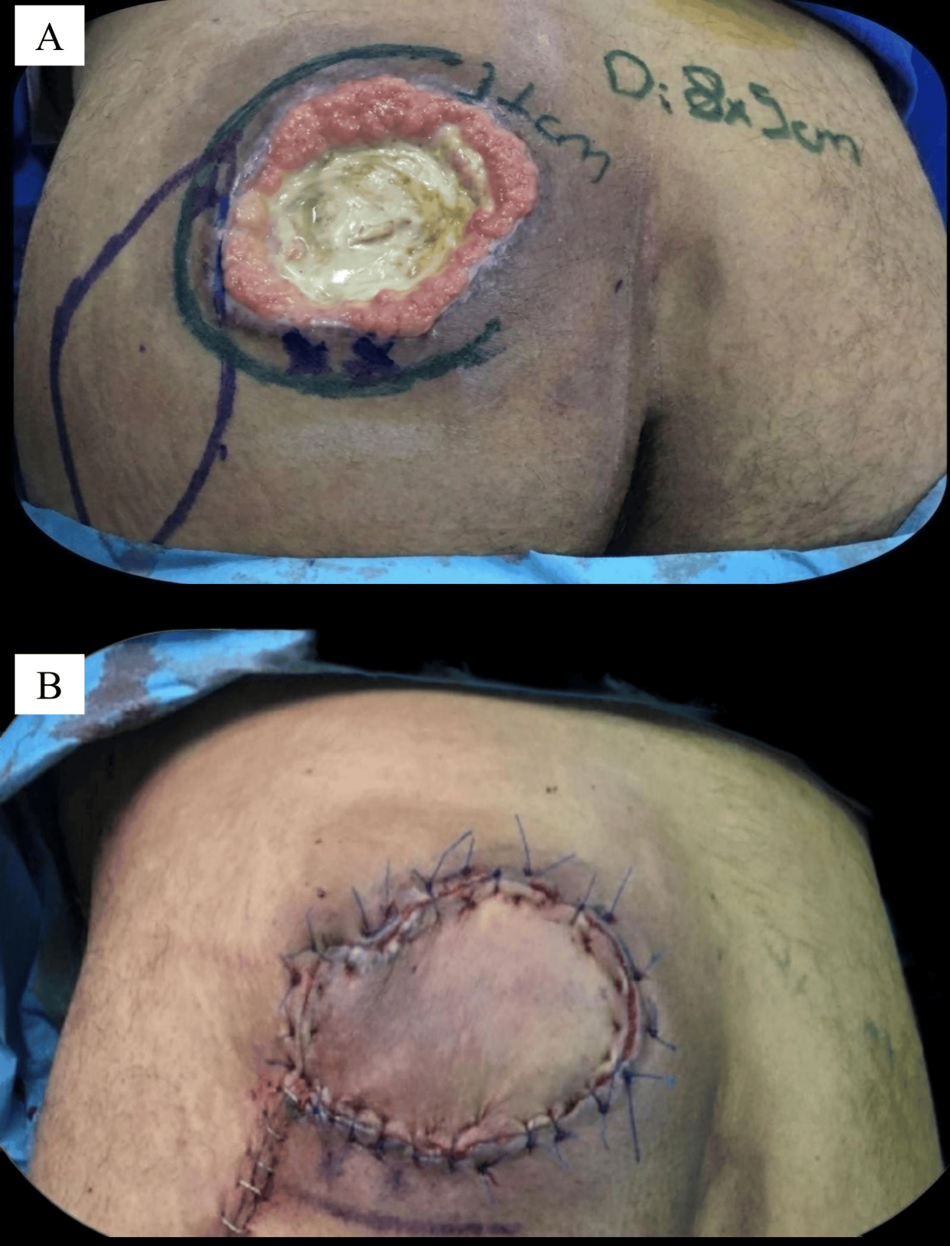
Surgical planning and immediate outcome of a superior gluteal artery perforator (SGAP) flap for a sacral pressure ulcer. (A) Preoperative presentation of a Stage IV sacral pressure ulcer with a documented defect size of 8 × 5 cm and a planned 3.5 cm surgical margin. The template design for the perforator-based flap transposition is explicitly mapped on the left gluteal region. (B) Immediate postoperative result following successful transposition and inset of the SGAP island flap into the sacral defect, demonstrating primary closure with surgical sutures and an optimized, tension-free skin approximation along the donor site line.

**Figure 4:**
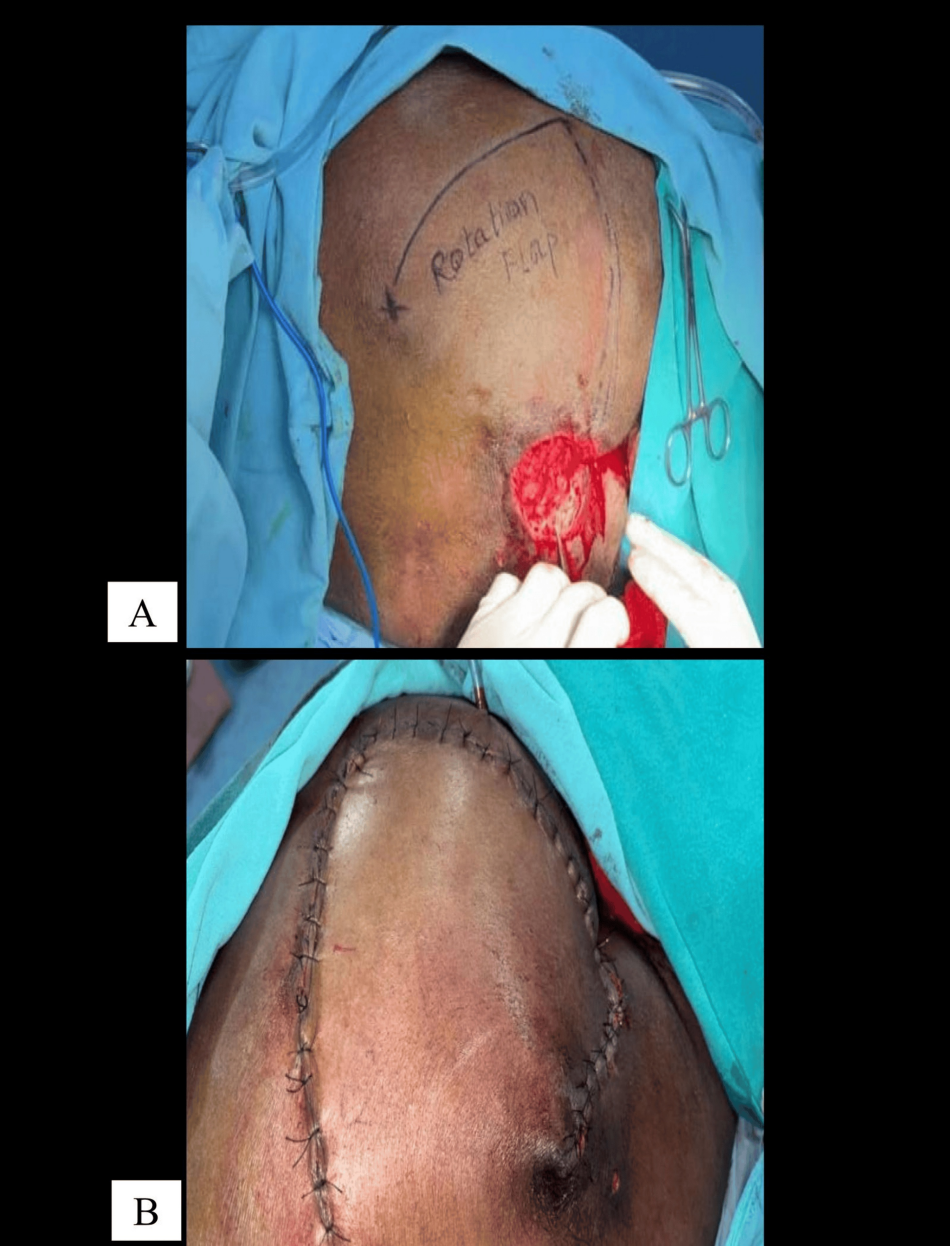
Intraoperative design and execution of a large gluteal rotation flap for a sacral defect. (A) Intraoperative surgical planning displaying the mapped semicircular incision line for a gluteal rotation flap adjacent to a freshly debrided sacral pressure ulcer bed. (B) Immediate postoperative result following successful clockwise flap transposition, demonstrating precise margins, tension-free alignment, and primary closure.

**Figure 5:**
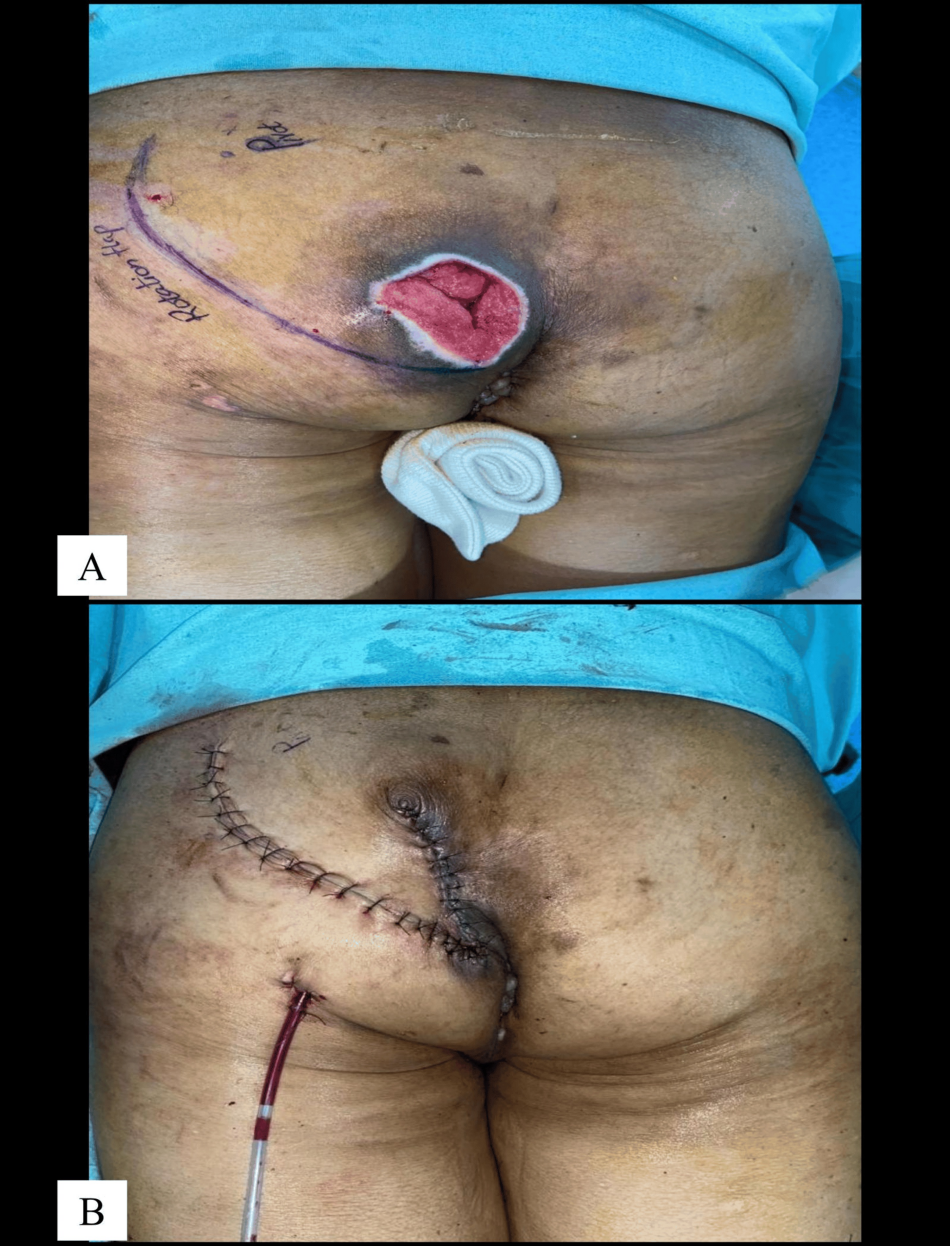
Reconstructive sequence of a large sacral pressure ulcer using a gluteal rotation flap. (A) Preoperative view of a wide, advanced Stage IV sacral pressure ulcer demonstrating heavy central slough and irregular, fibrotic margins. (B) Late postoperative review showing completely stable healing of the transposed gluteal rotation flap with well-matured scars and robust coverage over the sacral prominence. Drain in situ.

​Alternatively, when muscle preservation was paramount for sacral defects, a superior gluteal artery perforator (SGAP) flap was selected. Handheld Doppler ultrasonography was used preoperatively to identify and map the dominant SGAPs, assessing the advanced presentation exhibiting a deep central crater and fibrotic margins (Figure 2A and Figure 3A). An island flap template was designed over the chosen vascular axis matching the debrided defect dimensions, allowing successful suprafascial transposition, immediate inset into the sacral defect, and primary donor line approximation (Figure 3B), which resulted in robust soft tissue padding over the sacral prominence at late postoperative follow-up (Figure 2B). For large but relatively superficial sacral defects, gluteal rotation flaps provided an effective alternative. A wide, semicircular incision line was designed extending superiorly and laterally from the wound margin across the gluteal region (Figure 4A and Figure 5A). The resulting flap was raised with a broad base to ensure robust axial perfusion, rotated clockwise or counterclockwise into the debrided wound bed, and anchored securely over a drain in situ to ensure primary closure and well-matured scarring (Figure 4B and Figure 5B).

**Figure 6:**
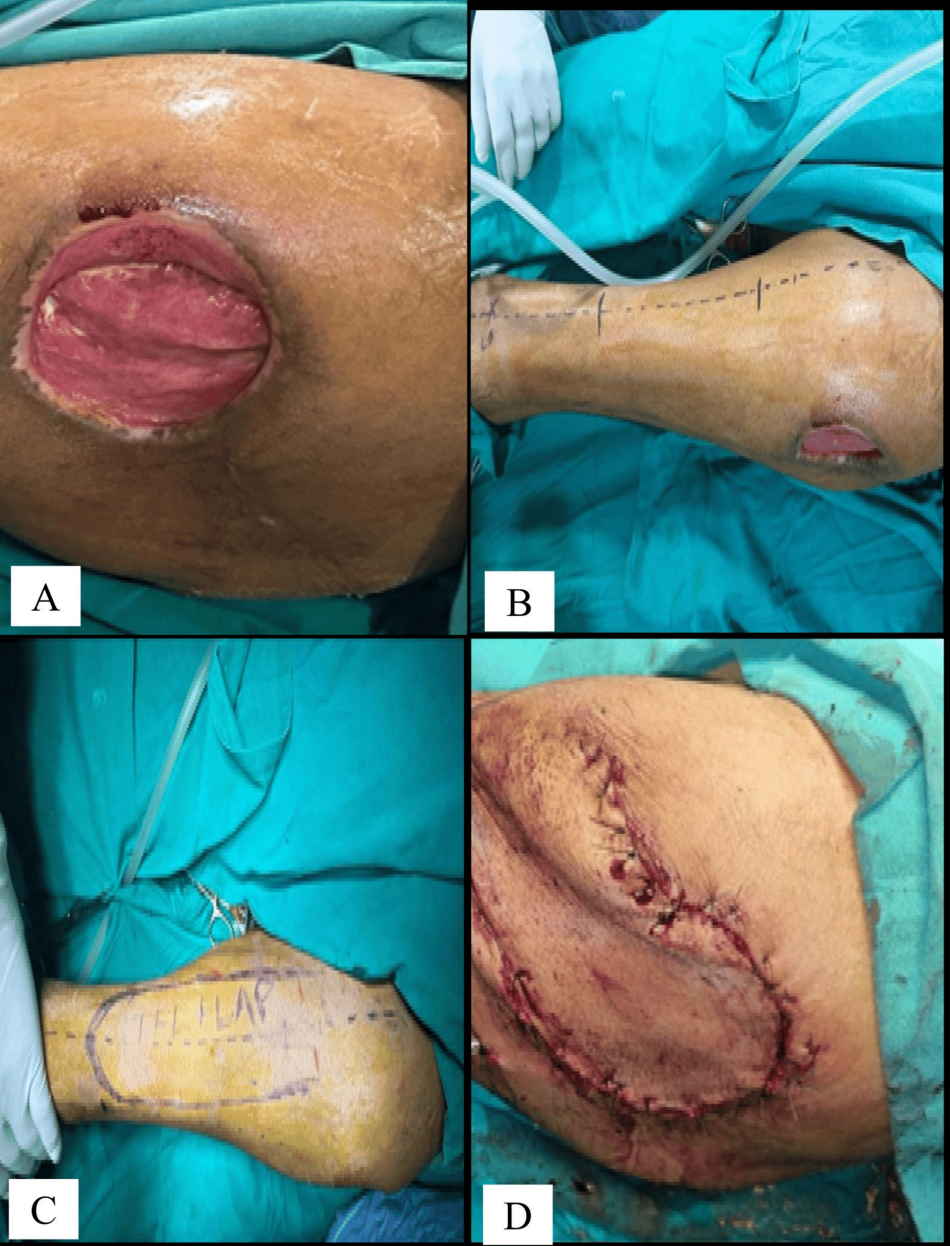
Comprehensive surgical stages of a trochanteric pressure ulcer reconstruction using a tensor fascia lata (TFL) flap. (A) Preoperative presentation of an advanced Stage IV pressure ulcer over the trochanteric region with exposed deep tissue. (B) Intraoperative view following radical surgical debridement of necrotic tissue and initial axis marking along the lateral thigh. (C) Definitive surgical planning and design of the TFL flap, with the margins clearly delineated on the skin surface prior to incision. (D) Immediate postoperative outcome displaying complete flap transposition, tension-free inset, and secure wound closure.

**Figure 7:**
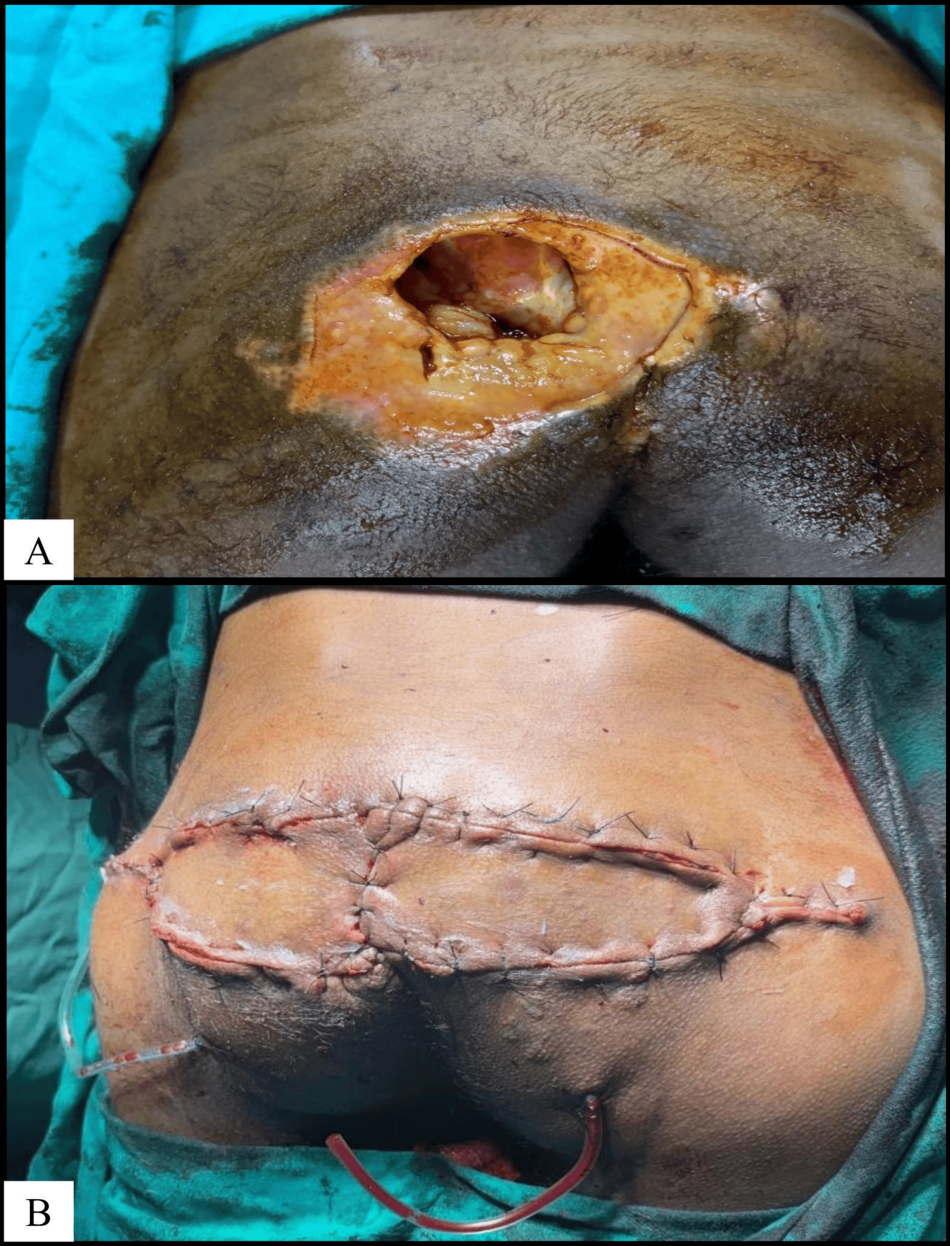
Bilateral V-Y advancement flap reconstruction for an advanced sacral pressure ulcer. (A) Preoperative clinical view of a large, undermining Stage IV sacral pressure ulcer exhibiting a deep cavity and exposed underlying tissue layers. (B) Immediate postoperative appearance after complete bilateral V-Y advancement flap transposition. The image shows the integrated "island" closure configuration over the sacrum with two closed-suction surgical drains placed in situ to prevent fluid accumulation.

For the exclusive management of trochanteric pressure ulcers, a TFL flap was employed. With the patient positioned laterally, the advanced stage of the lateral hip pressure ulcer was evaluated (Figure 6A). Following radical debridement, initial axial alignment markings were drawn along the lateral aspect of the thigh (Figure 6B). A longitudinal flap design was mapped between the anterior superior iliac spine and the lateral condyle of the femur, customized to match the dimensions of the debrided surgical defect (Figure 6C). The flap was elevated from distal to proximal, incorporating the fascia lata to protect the vascular pedicle originating from the lateral circumflex femoral artery, and the mobilized tissue island was transposed superiorly into the trochanteric defect to achieve a tension-free inset and secure primary wound closure (Figure 6D). For advanced undermining sacral cavities requiring robust, bilateral coverage, a complementary bilateral V-Y advancement approach was executed to obliterate extensive deep craters (Figure 7A) by advancing dual tissue islands into an integrated midline closure supported by deep suction drainage (Figure 7B). Following final closure across all techniques, patients were immediately transitioned onto specialized low-air-loss beds postoperatively to maintain strict pressure off-loading.

Outcome assessment

Postoperative outcomes were assessed during hospital stay and follow-up visits. A successful outcome was defined as complete flap survival with primary wound healing and absence of recurrence during follow-up. Postoperative complications, including seroma formation, wound dehiscence, partial flap necrosis, and abscess formation, were recorded. Factors associated with successful flap outcomes, such as adequate nutritional support, patient positioning, spasm control, bladder and bowel control, absence of active infection or osteomyelitis, and proper preoperative pressure off-loading, were also evaluated.

Statistical analysis

Data were analyzed using the Statistical Package for the Social Sciences (SPSS) (IBM SPSS Statistics for Windows, IBM Corp., Version 26.0, Armonk, NY). Descriptive statistics, including mean, standard deviation, frequency, and percentage, were used to summarize the data. Associations between patient characteristics, ulcer-related factors, and postoperative outcomes were evaluated using suitable statistical tests. A p-value of less than 0.05 was considered statistically significant.

## Results

A total of 20 patients with Stage III and Stage IV pressure ulcers underwent flap reconstruction during the study period. The mean age of the patients was 58.9 ± 10.6 years, with ages ranging from 45 to 75 years. Male predominance was observed with a male-to-female ratio of 1.5:1, consisting of 12 (60.0%) males and eight (40.0%) females. Sacral pressure ulcers were the most common ulcer type encountered (Table 1). The study population predominantly consisted of elderly male patients. Sacral ulcers accounted for half of all pressure ulcers, impacting 10 (50.0%) patients, and most ulcers were Stage IV lesions, found in 12 (60.0%) patients.

**Table 1:** Baseline demographic and ulcer characteristics of study participants.

Variable	Frequency (n = 20)	Percentage (%)
Gender		
Male	12	60.0
Female	8	40.0
Age group (years)		
45-55	6	30.0
56-65	8	40.0
>65	6	30.0
Mean age (years) - 58.9 ± 10.6		
Ulcer site		
Sacral	10	50.0
Trochanteric	5	25.0
Gluteal	2	10.0
Ischial	1	5.0
Other sites	2	10.0
Ulcer stage		
Stage III	8	40.0
Stage IV	12	60.0
Associated infection/osteomyelitis	3	15.0
Anemia present	5	25.0
Comorbidities present	6	30.0

Fasciocutaneous flaps and V-Y advancement flaps were the most commonly used reconstructive procedures.

Minor postoperative complications were observed in a few patients, but complete flap survival was achieved in all cases, with no recurrence (Table 2). The overall flap survival rate was 100%, while postoperative complications were observed in five (25.0%) patients. No recurrence was noted during the follow-up period. The majority of patients had uneventful healing. 

**Table 2:** Surgical procedures and postoperative outcomes.

Variable	Frequency (n = 20)	Percentage (%)
Type of flap used		
Bilateral V-Y advancement flap	5	25.0
Tensor fascia lata flap	3	15.0
SGAP flap	2	10.0
Rotation flap	2	10.0
Other fasciocutaneous flaps	8	40.0
Postoperative complications		
None	15	75.0
Seroma formation	2	10.0
Wound dehiscence	1	5.0
Partial flap necrosis	1	5.0
Abscess formation	1	5.0
Successful flap healing	20	100
Recurrence	0	0

Comorbidities were present in six (30.0%) patients, and anemia was observed among five (25.0%) patients with advanced ulcers (Table 1). Statistical analysis using Fisher's exact test demonstrated that poor nutritional status was significantly associated with postoperative complications, with 80.0% of the complicated cohort being malnourished compared to only 13.3% in the uneventful group (Fisher's exact p = 0.01; Table 3). Similarly, anemia was noted in 60.0% of patients with complications vs. 13.3% without complications, demonstrating statistical significance (Fisher's exact p = 0.04; Table 3). The presence of culture-positive infection or osteomyelitis carried an exceptionally strong correlation, present in 60.0% of patients with complications and entirely absent (0.0%) in those who healed uneventfully (Fisher's exact p = 0.002; Table3). Chronic ulcerations with a duration exceeding three months were found in 80.0% of the complicated group compared to 20.0% of the uncomplicated group, which was statistically significant (Fisher's exact p = 0.03; Table 3). Conversely, adequate postoperative pressure off-loading exerted a highly substantial protective effect against developing surgical morbidities, with 93.3% of patients who avoided complications utilizing optimized off-loading interfaces compared to only 20.0% in the complicated group (Fisher's exact p = 0.005; Table 3). Although uncontrolled diabetes mellitus and system-wide comorbidities were more prevalent in patients who developed complications (60.0% vs. 20.0%), this clinical trend did not reach the threshold for statistical significance (Fisher's exact p = 0.08; Table 3).

**Table 3:** Association of clinical factors with postoperative complications. An asterisk (*) indicates a statistically significant association (p < 0.05).

Variable	Complications present (n = 5)	No complications (n = 15)	Statistical test applied	Test statistic/p-value
Poor nutritional status	4 (80.0%)	2 (13.3%)	Fisher's exact test	0.01*
Anemia	3 (60.0%)	2 (13.3%)	Fisher's exact test	0.04*
Infection/osteomyelitis	3 (60.0%)	0 (0.0%)	Fisher's exact test	0.002*
Ulcer duration >3 months	4 (80.0%)	3 (20.0%)	Fisher's exact test	0.03*
Uncontrolled diabetes/comorbidities	3 (60.0%)	3 (20.0%)	Fisher's exact test	0.08
Adequate pressure off-loading	1 (20.0%)	14 (93.3%)	Fisher's exact test	0.005*

## Discussion

Pressure ulcers continue to be a major reconstructive challenge, particularly in elderly and immobilized patients with multiple comorbidities. In the present study, most patients were older, with a male predominance. Similar findings were reported by Duci et al., who observed a higher incidence of pressure ulcers among male patients due to increased rates of spinal cord injury and prolonged immobilization [9]. Sacral ulcers were the most common ulcers encountered in our study, followed by trochanteric ulcers, which is consistent with the observations of Thiessen et al., who reported the sacral region as the most vulnerable pressure point in bedridden patients [10]. The majority of ulcers in the present study were Stage IV ulcers, indicating advanced tissue destruction at presentation. Delayed presentation, prolonged immobilization, poor hygiene, and inadequate pressure off-loading may have contributed to the severity of ulcers. Similar observations were made by Black et al., who emphasized that advanced-stage pressure ulcers are frequently associated with delayed referral and poor preventive care [11].

Flap reconstruction remains the standard treatment for advanced pressure ulcers because it provides durable vascularized tissue coverage and adequate padding over bony prominences. In the present study, fasciocutaneous flaps and bilateral V-Y advancement flaps were commonly used and showed satisfactory outcomes. Yamamoto et al. also demonstrated favorable healing and low recurrence rates with fasciocutaneous flap reconstruction in pressure ulcer management [12]. Complete flap survival was achieved in all patients in the present study, with no recurrence observed during follow-up. The low recurrence rate may be attributed to meticulous debridement, proper infection control, adequate pressure off-loading, and careful postoperative rehabilitation. Postoperative complications were observed in a small proportion of patients, with seroma formation being the most common complication. Similar findings were reported by Foster et al., who identified seroma and wound dehiscence as common early postoperative complications after flap surgery [13]. Importantly, poor nutritional status, anemia, prolonged ulcer duration, and infection/osteomyelitis showed statistically significant association with postoperative complications in the present study. These findings are supported by Bauer et al., who reported that malnutrition and anemia adversely affect collagen synthesis, tissue oxygenation, and wound healing [14]. The presence of osteomyelitis and active infection significantly increased the risk of postoperative complications in our patients. Goodman et al. similarly reported that untreated infection and chronic osteomyelitis are major predictors of delayed healing and flap failure [15]. Adequate pressure off-loading demonstrated a protective effect against postoperative complications in the present study. Pressure redistribution and postoperative rehabilitation are known to reduce tissue stress and improve flap survival [16].

Overall, the findings of the present study highlight that successful flap reconstruction depends not only on surgical technique but also on optimization of patient-related and ulcer-related factors. A multidisciplinary approach, including nutritional support, infection control, rehabilitation, and long-term follow-up, plays a crucial role in improving flap survival and reducing complications. Therefore, while surgical reconstruction provides an immediate structural solution, long-term success is heavily contingent upon optimizing these systemic and mechanical patient factors.

There are certain limitations to this study that warrant acknowledgment, primarily its retrospective observational design and its execution within a single tertiary care center. The sample size of twenty patients is relatively small, which inherently limits the statistical power to detect subtle influences or rare complications across a wider variety of specialized flap designs. Additionally, because data collection relied on existing institutional medical records, variables such as long-term post-discharge compliance with strict off-loading regimens and precise home-based nutritional maintenance could not be continuously tracked or standardized. The follow-up period, while sufficient to document immediate postoperative flap survival and initial wound stabilization, was not long enough to establish true lifelong recurrence rates or late-stage structural breakdown of the reconstructed sites. Future multi-centric, prospective clinical studies with larger patient cohorts and extended, multi-year follow-up intervals are required to more comprehensively validate these preoperative risk factors and build institutional predictive protocols for advanced pressure ulcer management.

## Conclusions

Flap reconstruction is an effective surgical modality for the management of advanced Stage III and Stage IV pressure ulcers with satisfactory flap survival and low complication rates. Successful outcomes are influenced not only by the flap technique but also by patient-related factors such as nutritional status, anemia, infection control, and comorbidity management. Adequate pressure off-loading, proper wound bed preparation, and postoperative rehabilitation significantly contribute to improved healing and prevention of recurrence. A multidisciplinary approach with careful patient optimization and follow-up is essential for achieving favorable long-term outcomes in pressure ulcer reconstruction.
